# Tanshinone IIA attenuates psoriasis via Nrf2/HO-1 activation: Mechanistic insights from preclinical models

**DOI:** 10.1016/j.bbrep.2026.102606

**Published:** 2026-05-11

**Authors:** Xia Xu, Jingyao Liang, Maofang Huang, Quan Chen, Shijian Xiang, Xianhua Tang, Zhang Jingyue

**Affiliations:** a Department of Dermatology, Guangzhou Dermatology Hospital, Guangzhou, Guangdong, China; b Department of Clinical Laboratory, Guangzhou Dermatology Hospital, Guangzhou, Guangdong, China; c Department of Pharmacy, The Seventh Affiliated Hospital, Sun Yat-sen University, Guangzhou, Guangdong, China; d Department of Dermatology, Panyu Maternal and Child Care Service Centre of Guangzhou, Guangzhou, Guangdong, China; e Department of Clinical Laboratory, Eighth Affiliated Hospital of Guangxi Medical University, Guigang City People's Hospital, Guigang, Guangxi, China

**Keywords:** Tanshinone IIA, Nrf2 signalling, Psoriasis, Oxidative stress, Antioxidant therapy

## Abstract

**Background:**

Psoriasis is a chronic inflammatory skin disorder driven by oxidative stress and immune dysregulation. Tanshinone IIA, a bioactive compound from *Salvia miltiorrhiza*, exhibits antioxidant properties, but its role in treating psoriasis remains underexplored.

**Methods:**

Using H_2_O_2_-stimulated HaCaT keratinocytes and an imiquimod (IMQ)-induced murine psoriasis model, we investigated the therapeutic effects of tanshinone IIA. Key assays included Reactive Oxygen Species (ROS) detection, EdU/CCK8 proliferation analysis, real-time quantitative PCR (RT-qPCR), western blotting, and immunohistochemistry to evaluate nuclear factor erythroid 2-related factor 2 (Nrf2) activation, antioxidant protein expression, and histopathological changes.

**Results:**

Tanshinone IIA significantly suppressed TNF-α-induced HaCaT proliferation and ROS accumulation. Mechanistically, it promoted Nrf2 nuclear translocation and upregulated HO-1, SOD2, and NQO1 expression. In IMQ-treated mice, it reduced epidermal thickness, scaling, and inflammatory cytokines while enhancing antioxidant defences.

**Conclusion:**

Tanshinone IIA mitigates psoriasis via Nrf2/HO-1 activation and thus has therapeutic potential.

## Highlights

• Tanshinone IIA activates Nrf2/HO-1 signaling in psoriasis.
• Tanshinone IIA suppresses oxidative stress in IMQ-induced psoriasis.
• Reduces keratinocyte proliferation and ROS accumulation via Nrf2.
• Nrf2 knockdown abolishes Tanshinone IIA's antioxidant effects.

## 1 Introduction

Psoriasis is a chronic, relapsing, systemic inflammatory disease mediated by immune dysregulation and influenced by genetic and environmental factors. It affects approximately 2–3% of the global population and is characterized by epidermal keratinocyte hyperproliferation, inflammatory cell infiltration, and oxidative stress imbalance. Clinically, psoriasis presents as erythematous plaques with silvery scales and epidermal thickening, and it significantly impairs patients’ quality of life [1]. Traditional systemic therapies, such as cyclosporine, methotrexate, and acitretin, are associated with significant adverse effects, including hepatotoxicity and teratogenicity. Although biologics that target interleukin-17 (IL-17) (e.g. secukinumab) have demonstrated notable clinical efficacy, limitations persist. Some patients may exhibit suboptimal responses, and therapeutic effectiveness may diminish over prolonged treatment periods [2]. Furthermore, the long-term use of biologics is associated with infection risks and, in turn, escalating healthcare costs due to the need for rigorous monitoring [3,4]. The complexity of psoriasis arises from its intricate pathophysiology, which involves deregulated immune cell interactions and a cascade of inflammatory signals triggered by environmental cues in genetically susceptible individuals.

Inflammation and oxidative stress are pivotal mechanisms underlying the pathogenesis of psoriasis. These processes contribute to lipid peroxidation, DNA damage, and proinflammatory cytokine secretion, thereby exacerbating skin lesions and promoting disease progression [5,6]. Inadequate cellular antioxidant defences drive an imbalance in redox status, shifting the equilibrium towards oxidative stress; this has been hypothesized to be a fundamental driver of psoriatic manifestation [7].

Recent years have seen a surge of interest in identifying natural compounds with antioxidant properties that can mitigate oxidative stress and inflammation in psoriasis cases. Among these, tanshinone IIA, a major lipophilic constituent derived from the roots of *Salvia miltiorrhiza* (red-rooted salvia), has garnered attention for its diverse pharmacological activities and has demonstrated antioxidant, antiproliferative, antiangiogenic, and anti-inflammatory effects in various studies [8,9]. In particular, its therapeutic potential against skin disorders is likely due to its ability to inhibit VEGFA expression and modulate cellular signalling pathways [8]. Tanshinone IIA has also been shown to perform a protective function against apoptosis in different disease models, including traumatic spinal cord injury, by reducing oxidative stress and inflammatory responses [10].

Research into the mechanisms underpinning the therapeutic actions of tanshinone IIA has unveiled its interaction with the Nrf2/HO-1 signalling pathway. Upon activation by oxidative stress, Nrf2, a master regulator of cellular antioxidant defences, orchestrates the transcription of genes that encode antioxidant proteins and detoxify enzymes. This activation involves Nrf2's translocation to the nucleus, where it binds to antioxidant response elements (AREs), leading to the upregulation of proteins such as HO-1, NADP(H) quinone oxidoreductase-1 (NQO1), glutathione S-transferase, and aldo-keto reductase 1 (AKR1) [11,12]. Despite the established role of Nrf2/HO-1 in oxidative stress, its modulation by natural compounds remains underexplored in psoriasis cases. Given the significant impact of oxidative stress and inflammation on psoriasis and the emerging evidence supporting the antioxidant and anti-inflammatory properties of tanshinone IIA, there is compelling rationale for investigating this compound's potential protective effects against psoriasis via the Nrf2/HO-1 pathway. The present study was conducted to address the aforementioned gap by investigating tanshinone IIA's dual antioxidant and anti-inflammatory effects via Nrf2/HO-1. The specific aim of the study was to elucidate the impact of tanshinone IIA on Nrf2 activation and subsequent antioxidant responses through in vitro and in vivo experiments, thereby contributing to the understanding of tanshinone IIA's therapeutic mechanisms and identifying a novel therapeutic strategy for psoriasis.

## 2 Materials and Methods

### 2.1 Cell culture and reagents

The Immortalized Human Epidermal Keratinocytes (HaCaT) (Shanghai Fuheng Biotechnology Co.,Ltd, catalog number FH0186) was cultivated in Dulbecco's Modified Eagle Medium (DMEM, Gibco) supplemented with 10% foetal bovine serum at a temperature of 37 °C and in an atmosphere of 5% CO2. The cell line was authenticated by short tandem repeat (STR) profiling within the last three years and routinely tested negative for mycoplasma contamination.Cells that achieved a confluence of 40–60% were stimulated with H_2_O_2_ (10 μM). ELISA kits were sourced from Roche Diagnostics (Indianapolis, IN). Imiquimod was supplied by Sigma-Aldrich (USA). Both tanshinone and tanshinone IIA were purchased from Sigma-Aldrich (MedChemExpress, USA).

### 2.2 Imiquimod-induced psoriasis model

Twenty-four male C57BL/6 J mice (6-8 weeks old) were purchased from GemPharmatech Laboratory (Nanjing, China) and maintained on a standard diet with water ad libitum. Mice were randomly allocated into four groups (n = 6 per group).All in vivo experiments were conducted in accordance with the guidelines of the Laboratory Animal Research Committee and the Institutional Animal Care and Use Committee.The experimental protocol was approved by animal Ethics Committee of the Guangzhou Dermatology Hospital committee (Approval No:B202409-14).To induce psoriasis, eight-week-old C57BL/6 mice were randomly assigned to four groups – (1) vehicle (Phosphate Buffered Saline, PBS), (2) IMQ model (topical IMQ), (3) IMQ + tanshinone (50 mg/kg oral), and (4) IMQ + tanshinone IIA (5 mg/kg oral) – and then pretreated with or without imiquimod on their backs for three consecutive days. The experimental protocol was performed according to the method described by Guo et al. [8]. Subsequently, tanshinone or tanshinone IIA was orally administered.

### 2.3 Enzyme-linked immunosorbent assay

Following the application of imiquimod, the mice were orally administered the PBS, tanshinone, or tanshinone IIA. Blood samples were collected via cardiac puncture 90 min after imiquimod administration. The samples were centrifuged at 2000 g for 20 min to obtain serum, which was then diluted 10-fold with ELISA diluent solution (eBioscience, San Diego, CA, USA). The concentrations of serum tumour necrosis factor-α (TNF-α), IL-17, and NQO1 were determined using an ELISA kit (eBioscience) according to the manufacturer's protocol. Absorbance was measured at 450 nm and 540 nm using a BMG PheraStar plate reader, and the concentrations were extrapolated from standard curves.

### 2.4 Real-time quantitative PCR (RT-qPCR) assay

Total tissue RNA was extracted using an RNeasy mini kit (QIAGEN, Valencia, CA, USA) according to the manufacturer's instructions. One microgram of total RNA was reverse-transcribed using Maxime RT PreMix (Bioneer, Daejeon, Korea) with oligo-dT15 primers. RT-qPCR was performed using SYBR Green 1 (Roche) and normalized to GAPDH gene expression. The primers used are listed in Supplementary Table S1.

### 2.5 Western blot assay

The tissue samples for the Western blot analysis were prepared using a lysis buffer. The protein concentrations of the cell lysates were quantified using BCA reagent (Beyotime). Subsequently, 20 μg of protein from each sample was resolved on a 12% SDS-polyacrylamide gel by electrophoresis and transferred onto a polyvinylidene difluoride membrane. The membrane was then incubated with the appropriate primary antibodies (see Supplementary Table S2 for dilution parameters), followed by horseradish peroxidase-conjugated anti-rabbit or anti-mouse secondary antibodies. Protein bands were visualized using an enhanced chemiluminescence detection system (Amersham Biosciences, Piscataway, NJ, USA), and images were captured using the Image J analyser.

### 2.6 Histological analyses

To evaluate histopathological alterations, skin samples were fixed in 4% paraformaldehyde for 48 h, embedded in paraffin, and sectioned into 5 μm thick slices. These paraffin sections were stained with haematoxylin and eosin (H&E) and examined under an optical microscope (Olympus D72, Olympus Corporation, Tokyo, Japan).

### 2.7 Immunohistochemical (IHC) analysis

An IHC analysis was conducted as previously reported [13]. After antigen retrieval, the tissue sections were incubated with 5% normal goat serum for 30 min and then incubated for 2 h with specific antibodies (see Supplementary Table S2 for dilution parameters). Following this, the sections were incubated with a biotinylated secondary antibody and developed using a 3,3′-diaminobenzidine staining kit (DAKO, Carpinteria, CA, USA). Counterstaining was performed with haematoxylin.

### 2.8 EdU proliferation assay

HaCaT cells were plated in quadruplicate in 24-well plates at a density of 1 x 10^5 cells per well and incubated for 24 h. The cells were then treated with tanshinone IIA (5 μM) and H_2_O_2_ (10 μM). Cell proliferation was assessed using an EdU cell proliferation assay kit (Ribobio, Guangzhou, China) 48 h post treatment. The cells were exposed to 20 μM of EdU for 2 h prior to fixation and permeabilization. The cell nuclei were stained with 100 μl of Hoechst 33342 (5 μg/ml) for 10 min. The proportion of EdU-positive cells in each group was determined using fluorescence microscopy (Olympus, Tokyo, Japan) in five randomly selected fields of view.

### 2.9 Cell viability assay

HaCaT cells were treated with tanshinone IIA (5 μM) and H_2_O_2_ (10 μM) for 48 h and then plated in quadruplicate in 96-well plates at a density of 1 x 10^3 cells per well. The cells were incubated with CCK8 for 2 h. Absorbance was then measured at a wavelength of 450 nm using a multi-microplate multimode reader (Promega, USA).

### 2.10 Cell apoptosis by flow cytometry

HaCaT cells were subjected to tanshinone IIA (5 μM) and H2O2 (10 μM) for 8 h and subsequently trypsinized. A total of 1 x 10^6 cells from each treatment group were fixed with 4% paraformaldehyde (Sigma-Aldrich). Single-cell suspensions were stained with FITC-labelled Annexin V and propidium iodide, and the stained cells were analysed using an FACSCalibur flow cytometer (BD Biosciences, San Jose, CA, USA) to determine their apoptosis levels.

### 2.11 Immunofluorescence staining

For the immunofluorescence analysis, HaCaT cells were incubated overnight at 4 °C with the primary antibody anti-Nrf2 (rabbit monoclonal; 1:100, Abcam, Cambridge, UK). The cells were then washed three times with PBS and stained with FITC-tagged goat anti-rabbit (1:500) secondary antibodies. Nuclear counterstaining was performed using 4,6-diamidino-2-phenylindole (DAPI). Images were captured using a fluorescence microscope (Olympus, Tokyo, Japan) and analysed using Olympus FluoView™ FV1000 software.

### 2.12 Statistical analysis

The data obtained were presented as mean values ± standard errors for multiple independent experiments. Statistical analyses were conducted using SPSS software (version 22.0, SPSS Inc., Chicago, IL, USA). Significant differences were identified through a one-way analysis of variance (ANOVA), followed by Dunn's post-hoc test. The level of statistical significance was set at *P* < 0.05.

## 3 Results

### 3.1 Tanshinone IIA attenuated psoriasis in the mouse model

**Fig. 1:**
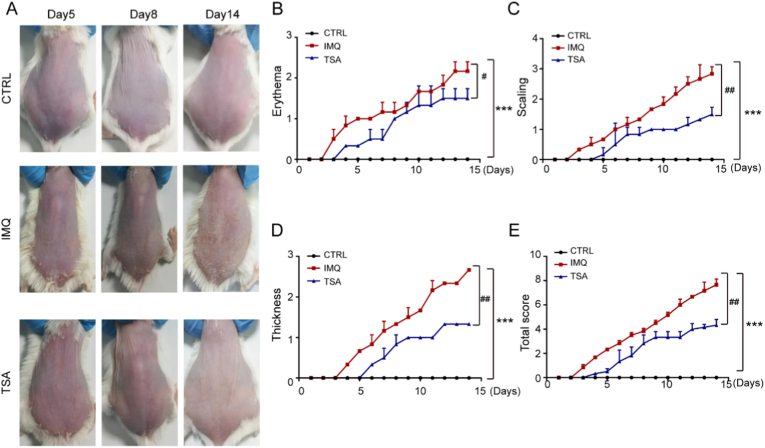
Tanshinone IIA improves psoriasis in model mice and reduces pathological changes. **A.** Psoriasis model mice in each group. **B–D.** Analysis results show that tanshinone IIA can decrease scaling, erythema, and skin inflammation thickness. Data are presented as the mean ± standard deviation values for at least three repeat experiments; n = 6.Data are presented as mean ± SD. ∗∗∗*P* < 0.001 vs the control group. ^#^*P* < 0.05,^##^*P* < 0.01 vs the TSA group,TSA, tanshinone IIA; IMQ, imiquimod; CTRL, Control.

**Fig. 2:**
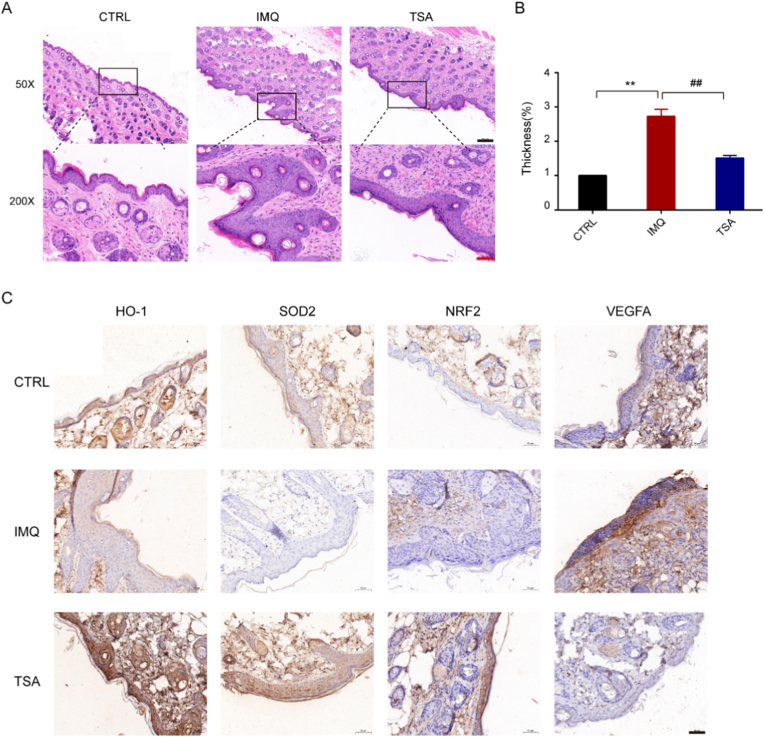
Tanshinone IIA attenuates imiquimod-induced psoriasis in model mice. The animals and treatments used are described under Materials and Methods. **A.** Representative H&E staining of skin tissue sections (100 × magnification). **B.** Statistical analysis shows that tanshinone IIA preserved skin inflammation. **C.** Effects of tanshinone IIA on HO-1, SOD2, and Nrf2 expression in the skin tissues of psoriasis mice, as determined by immunohistochemistry (200 × magnification). Scale bar = 100 μm.Data are presented as mean ± SD. ∗∗*P* < 0.01 vs the control group.^##^*P* < 0.01 vs the TSA group.

To assess the therapeutic potential of tanshinone IIA in psoriasis cases, we employed a mouse model of psoriasis induced by topical application of IMQ. One group of mice received a single intragastric administration of 5 mg/kg tanshinone IIA. Psoriasis severity was evaluated based on the Psoriasis Area and Severity Index (PASI) score on day 15**,** and phenotypic presentations of the back skin of the mice were captured (Fig. 1A). A gross morphological analysis corroborated these findings, showing attenuated erythema, scaling, and inflammation thickness in the tanshinone IIA-treated mice (Fig. 1A). The findings indicate that tanshinone IIA has an antipsoriatic effect (**P < 0.05;** Fig. 1B–D). A histological examination via HE staining revealed that IMQ induced epidermal hyperplasia and acanthosis in the epidermis, consistent with the pathological characteristics of psoriasis lesions. Notably, tanshinone IIA administration significantly alleviated these pathological changes, as evidenced by a decrease in the thickness of the epidermis layer (Fig. 2A and B). Furthermore, immunohistochemical staining demonstrated an increase in the number of positive HO-1, SOD2, and Nrf2 cells in the IMQ-induced psoriasis model following tanshinone IIA administration (Fig. 2C). Additionally, throughout the treatment period, mice in the tanshinone IIA-treated group did not exhibit any significant changes in body weight, food intake, or general behavior compared to the vehicle control group (data not shown). No gross pathological abnormalities were observed in major organs (liver, kidney) upon necropsy, suggesting that the 5 mg/kg dose was well-tolerated in this subacute setting. However, future studies should include dedicated subacute toxicity tests with histopathological analysis of major organs and measurement of serum biomarkers (e.g., ALT, AST, creatinine) to fully evaluate the safety of long-term use.These results suggest that tanshinone IIA exerts its antipsoriatic effects, at least in part, by enhancing antioxidant pathways.

### 3.2 The antioxidant effect of tanshinone IIA on IMQ-induced psoriasis in vivo

**Fig. 3:**
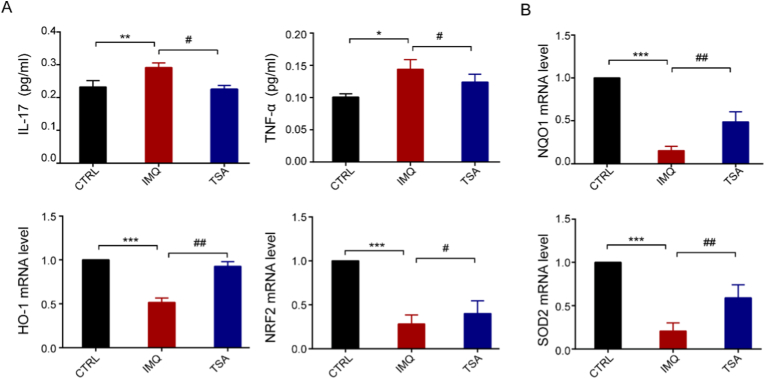
Tanshinone IIA promotes the mRNA expression levels of NRF2 and HO-1 in the IMQ-induced psoriasis models. **A.** Effects of tanshinone IIA on inflammatory factors IL-17 and TNF-α in the blood samples of psoriasis mice, determined using an ELISA kit. **B.** Relative mRNA expression levels of Nrf2, SOD, NQO1 and HO-1 in the skin tissues, as determined by RT-qPCR analyses. Data are presented as mean ± standard deviation values for three repeat experiments; n = 6. Data are presented as mean ± SD.∗*P*< 0.05, ∗∗*P*< 0.01, ∗∗∗*P* < 0.001 vs the control group. ^#^*P* < 0.05,^##^*P* < 0.01 vs the TSA group.

The study findings revealed that tanshinone IIA significantly inhibited the protein expression levels of IL-17 and TNF-α, which are involved in oxidative stress events. (Fig. 3A). To investigate whether this inhibitory effect was due to the transcriptional activation of Nrf2, we analysed the mRNA expression levels of Nrf2, SOD2, NQO1, and HO-1. Consistent with our hypothesis, tanshinone IIA significantly promoted the expression of these genes. Since Nrf2 regulates antioxidative enzymes such as HO-1 and SOD2, we verified the effect of tanshinone IIA on HO-1 and SOD2 protein expression in IMQ-stimulated mice. The results showed that tanshinone IIA strongly facilitated HO-1 and SOD2 expression in a dose-dependent manner (Fig. 3B).

### 3.3 Tanshinone IIA induced toxicity in HaCaT cells

**Fig. 4:**
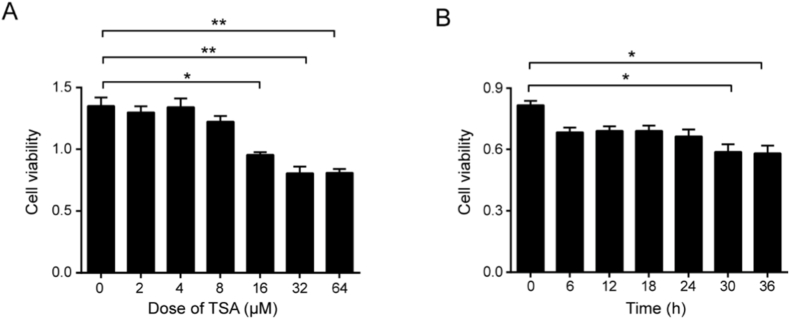
Tanshinone IIA induces toxicity in HaCaT cells. **A.** Effects of a concentration gradient of tanshinone IIA on HaCaT cells, determined using a CCK8 kit. **B.** Effects of a time gradient of tanshinone IIA on HaCaT cells, determined using a CCK8 kit. Data are presented as the mean ± standard deviation values for three repeat experiments; n = 6. Data are presented as mean ± SD.∗*P*< 0.05, ∗∗*P* < 0.01, vs the control group.

To evaluate the cytotoxicity of tanshinone IIA in HaCaT cells, we analysed its dose- and time-dependent effects using a CCK8 assay. As shown in Fig. 4A, after 2 h of treatment, only the highest concentration (16 μM) significantly reduced cell viability (*P* < 0.05) compared to the controls, whereas concentrations ≤8 μM did not induce significant toxicity. However, when exposure was extended to 6 h (Fig. 4B), even 5 μM of tanshinone IIA significantly decreased viability (*P* < 0.05). These results demonstrate that tanshinone IIA induces cytotoxicity in HaCaT cells in a dose- and time-dependent manner, highlighting a relatively narrow therapeutic window in vitro that necessitates careful dose selection for in vivo studies.

### 3.4 The antioxidant effect of tanshinone IIA on IMQ-induced psoriasis in vitro

**Fig. 5:**
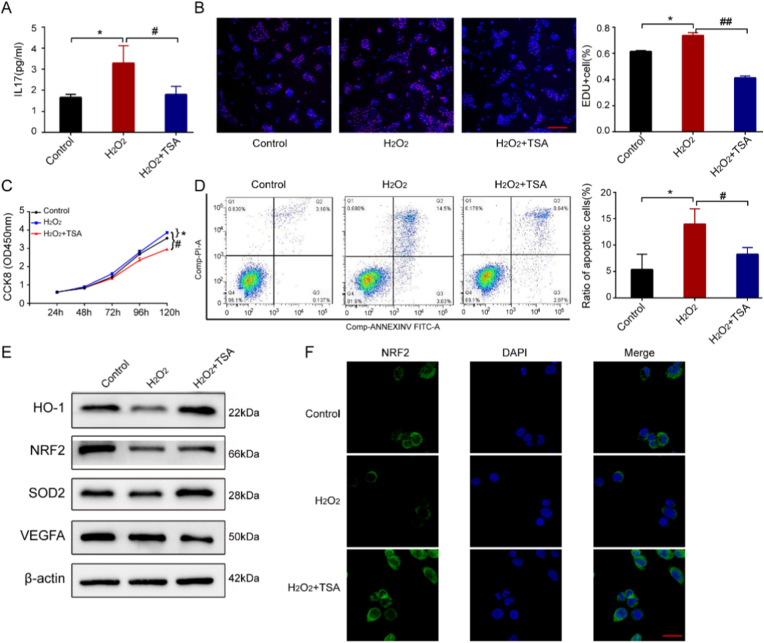
Pro-oxidant effects of tanshinone IIA on HaCaT cells. The cells were pretreated with 5 μM tanshinone IIA for 1 h prior to exposure to a 10 μM concentration of H_2_O_2_ for 48 h. **A.** Effects of tanshinone IIA on inflammatory factors IL-17 in HaCaT cells, determined using an ELISA kit. **B and C.** Effects of tanshinone IIA on HaCaT cell proliferation, as determined by EdU and CCK8 analyses; scale bar = 100 μm. **D.** The percentage of apoptotic cells determined by a flow cytometry analysis of annexin V-FITC binding. **E.** Nrf2 and Nrf2-regulating protein levels measured by western blotting. **F.** Representative pictures of HaCaT cells treated with tanshinone IIA and H_2_O_2_, analysed by immunofluorescence staining; scale bar = 200 μm.Data are presented as mean ± SD.∗*P* < 0.05 vs the control group. ^#^*P* < 0.05,^##^*P* < 0.01 vs the TSA group.

To investigate the impact of tanshinone IIA on cell growth, HaCaT cells were stimulated with H_2_O_2_. The cytokine IL-17 plays a crucial role in oxidative stress. Therefore, we investigated the effects of tanshinone IIA on the mRNA expression of IL-17 in HaCaT cells. The results showed that tanshinone IIA significantly inhibited IL-17 expression (Fig. 5A). Cell proliferation was monitored using EdU and CCK8. As anticipated, H_2_O_2_ significantly increased HaCaT cell proliferation. However, 10 μg/ml tanshinone IIA treatment dramatically decreased the growth of H_2_O_2_-stimulated HaCaT keratinocytes (*P* < 0.001) (Fig. 5B and C). The results of a flow cytometry analysis showed that H2O2 induced apoptosis, but tanshinone IIA treatment significantly reduced the number of apoptotic cells (Fig. 5D). These results suggest that tanshinone IIA can inhibit cell growth and reduce apoptosis in H_2_O_2_-stimulated HaCaT cells.

Further investigations of the mechanisms underlying the antioxidant effects of tanshinone IIA revealed that the levels of HO-1 and SOD2, both antioxidant proteins regulated by Nrf2, were significantly upregulated in the presence of tanshinone IIA, regardless of previous stimulation with proinflammatory cytokines. Tanshinone IIA displayed divergent effects on VEGFA expression (Fig. 5E). We also examined the expression and localization of Nrf2 under H_2_O_2_ stimulation. The findings showed that H_2_O_2_ decreased Nrf2 expression, but tanshinone IIA treatment significantly elevated Nrf2 accumulation in the nuclei and reduced its expression in the cytoplasm (Fig. 5F). This indicates that tanshinone IIA may promote the nuclear transfer of Nrf2.

### 3.5 Nrf2 knockdown revealed its central role in tanshinone IIA-mediated regulation of HO-1, SOD2, VEGFA, and reactive oxygen species (ROS)

**Fig. 6:**
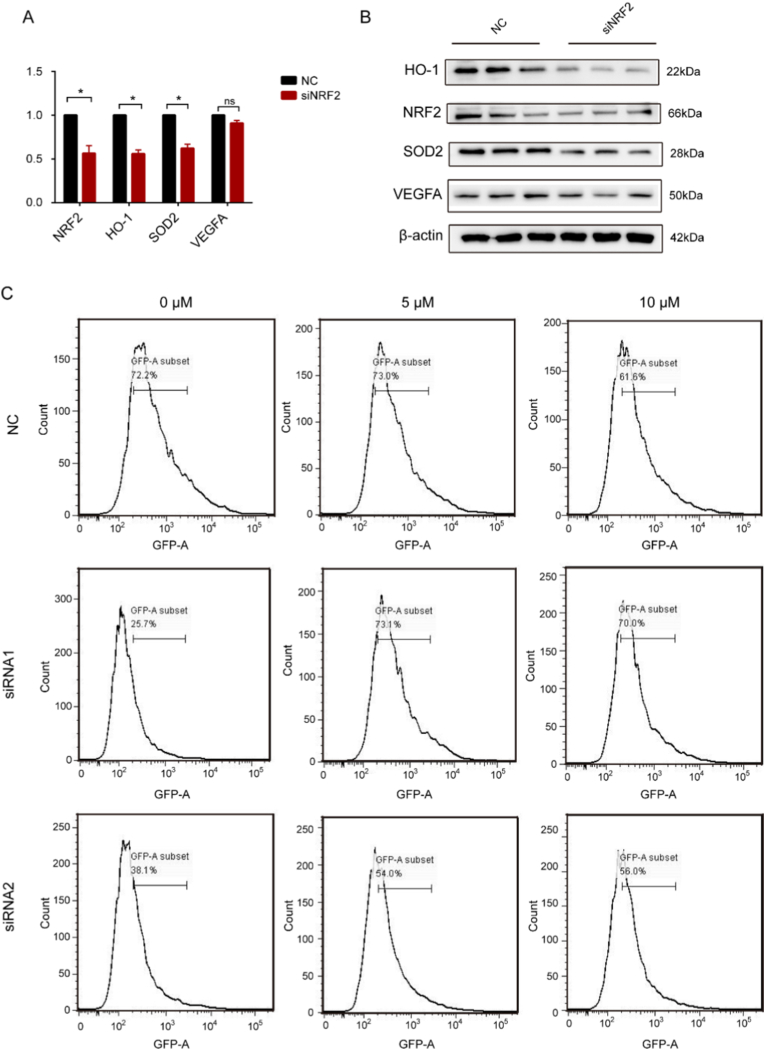
The effects of tanshinone IIA on HaCaT cells after NRF2 knockdown. **A.** qRT-PCR assessment of Nrf2 knockdown efficiency in HaCaT cells. **B.** Representative protein expression levels of Nrf2, SOD, HO-1, and VEGFA in HaCaT cells treated with tanshinone IIA after Nrf2 knockdown, as determined by western blotting. **C.** Flow cytometry analysis of ROS levels in HaCaT cells treated with tanshinone IIA after NRF2 knockdown.Data are presented as mean ± SD.∗*P* < 0.05 vs the control group.

**Fig. 7:**
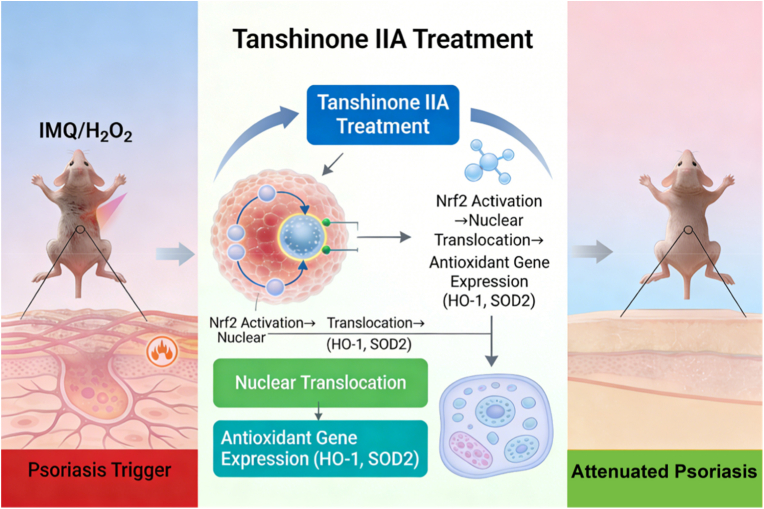
Schematic diagram of the molecular mechanism by which Tanshinone IIA attenuates psoriasis.

We treated HaCaT cells with tanshinone IIA and subsequently interfered with Nrf2 expression. The expression of HO-1 decreased after Nrf2 knockdown by siRNA, suggesting that tanshinone IIA regulates HO-1 expression through the Nrf2 signalling pathway (Fig. 6A). Further, the protein levels of SOD2 and VEGFA were both significantly downregulated after Nrf2 interference in the presence of tanshinone IIA (Fig. 6B). Since ROS production plays a critical role in oxidative stress, we measured the intracellular ROS levels and observed a significant increase after Nrf2 knockdown (Fig. 6C). Fig. 7 presents a schematic diagram of the molecular mechanism by which tanshinone IIA attenuates psoriasis.

## 4 Discussion

The findings of this study highlighted the protective effects of tanshinone IIA against psoriasis and its ability to treat oxidative damage induced by imiquimod. These effects appeared to be mediated by HO-1 induction, which is regulated by Nrf2. NRf2 is a transcription factor responsible for inducing HO-1, NQO1, glutathione S-transferase and others.

Tanshinone is an herbal drug extracted from the dried root of *Salvia miltiorrhiza* Bunge, which is used either alone or in combination with other herbal materials to clinically prevent or treat a variety of diseases in China and other countries. Tanshinone IIA, a water-soluble component, is one of the key constituents of *Salvia miltiorrhiza* [14] and has been found to possess anti-inflammatory, antiapoptotic, and antioxidant properties [[15], [16], [17]]. We evaluated the therapeutic effects of tanshinone IIA in a mouse model of psoriasis and found that it reduced skin erythema and swelling and promoted the recovery of skin lesions. These results indicate that tanshinone IIA has a significant protective effect against psoriasis.

Our findings are consistent with those of a previous study on the protective effects of tanshinone IIA in oxidative stress processes, which demonstrated that tanshinone IIA could inhibit the release of IL-17 [18]. Other studies involving in vivo experimental models have shown that tanshinone IIA can inhibit the production of ROS induced by TNF-α and interleukin-1β (IL-1β) [19,20]. Using the HaCaT cell line under H_2_O_2_ exposure, we demonstrated that the antioxidant activity of tanshinone IIA involves the NRF2 and HO-1 pathways and that its antioxidant protective effects include the reduction of oxidative stress.

The Nrf2/HO-1 pathway acts as a pivotal regulatory hub in psoriasis, coordinating integrated antioxidant and anti-inflammatory defenses. Psoriatic triggers like imiquimod (IMQ) disrupt redox balance and amplify inflammatory responses, while bioactive agents including Tanshinone IIA, propofol, gentiopicroside (GPS), and rutin modulate Keap1 or directly activate Nrf2. For instance, propofol has been reported to ameliorate psoriasiform lesions through KEAP1/Nrf2/HO-1 pathway activation [21]. Similarly, GPS has been shown to regulate the Keap1-Nrf2 pathway and inhibit keratinocyte activation in psoriatic mouse models [22]. Rutin induces Nrf2 nuclear translocation and upregulates downstream effectors (HO-1, SOD2, NQO1), which scavenge reactive oxygen species (ROS), suppress proinflammatory cytokines (IL-6, IL-17A, TNF-α), and inhibit keratinocyte hyperproliferation and immune cell infiltration [23]. Our findings with tanshinone IIA align with this growing body of evidence, demonstrating that its therapeutic effects are also mediated through Nrf2 nuclear translocation and the subsequent upregulation of its downstream targets HO-1, SOD2, and NQO1. While compounds like curcumin and sulforaphane are well-documented Nrf2 activators, a unique advantage of tanshinone IIA, as a major lipophilic constituent of *Salvia miltiorrhiza*, may lie in its specific pharmacological profile and traditional use in combination therapies, which warrants further comparative investigation. On the other hand, HO-1 and NQO-1 are recognized for their neuroprotective and anti-neuroinflammatory roles in the central nervous system (CNS), suggesting their potential as therapeutic targets for neuroinflammatory disorders [24,25]. HO-1 induction acts as the rate-limiting step in heme catabolism. During this process, free heme is oxidized to produce ferrous iron and biliverdin; subsequent reactions generate ferritin, carbon monoxide, and bilirubin [26]. HO-1 and its metabolic byproducts demonstrate antioxidant and anti-inflammatory activities. The expression of HO-1 and NQO-1 is regulated through the transcription factor Nrf2. This occurs via antioxidant response elements (AREs) in their gene promoters, which facilitate Nrf2 binding and the transcriptional activation of downstream genes involved in antioxidant and anti-inflammatory responses [27,28]. Therefore, targeting the Nrf2/HO-1 pathway has been explored as an approach to address neuronal damage linked to neuroinflammatory conditions. Further, the gene silencing of Nrf2 has been shown to exaggerate the inflammatory response and impair HO-1 induction, in turn aggravating neighbouring neuronal damage. As Nrf2/ARE plays a regulatory role in neuroinflammatory responses, it is a promising therapeutic target for the treatment of neurodegenerative diseases [29,30]. NF-κB is known to be a key driver of psoriatic inflammation, so future investigations should explore whether tanshinone IIA's effects on Nrf2 also modulate NF-κB activity.

Tanshinone IIA exerts its antioxidant effects via HO-1 regulation. HO-1 is a critical antioxidant enzyme that plays a central role in mitigating oxidative damage. Our results indicate that tanshinone IIA can prevent H_2_O_2_-induced oxidative stress through HO-1. This pathway is regulated by Nrf2, a neuroprotective transcription factor that modulates multiple genes that encode HO-1, SOD2, and ROS. Increased ROS levels in cells exacerbate psoriasis. The protective mechanism of tanshinone IIA involves reducing proinflammatory and oxidative mediators, and the effects are mediated through the Nrf2/HO-1 pathway. The regulatory role of HO-1 may explain how tanshinone IIA alleviates the effects of psoriasis in mice and highlights the potential role of tanshinone IIA in treating psoriasis-related diseases.

Furthermore, this study primarily focused on the role of tanshinone IIA in keratinocytes. Psoriasis pathogenesis is complex and involves intricate crosstalk between keratinocytes and infiltrating immune cells, such as T cells and dendritic cells, which constitute the inflammatory immune microenvironment. The effect of tanshinone IIA on Nrf2 activation within these specific immune cell populations, and how modulation of the Nrf2 pathway in one cell type might affect the behavior of others, was not explored. Future investigations should utilize co-culture systems or immune cell-specific Nrf2 knockout mouse models to dissect the role of tanshinone IIA in modulating the broader immune microenvironment in psoriasis.

## Consent for publication

Not applicable.

## Funding

This research was funded by the Guangzhou schools (institutes) and enterprises jointly funded special projects (SL2024A03J01122), and Scientific Research Project of Guangdong Provincial Administration of Traditional Chinese Medicine (No. 20261302), and it was also funded by the 10.13039/501100001809National Natural Science Foundation of China (No. 82560395) and the Guangxi Natural Science Foundation (No. 2024GXNSFBA010056).

## CRediT authorship contribution statement

**Xia Xu:** Writing – original draft. **Jingyao Liang:** Methodology. **Maofang Huang:** Resources. **Quan Chen:** Data curation. **Shijian Xiang:** Data curation. **Xianhua Tang:** Methodology. **Zhang Jingyue:** Funding acquisition, Supervision.

## Declaration of competing interest

The authors declare no potential conflicts of interest regarding this study.

## Data Availability

The data that support the findings of this study are available from the corresponding author upon reasonable request.
