# Supplementary material for: Tanshinone IIA attenuates psoriasis via Nrf2/HO-1 activation: Mechanistic insights from preclinical models
Source: Biochem Biophys Rep. 2026 May 11;46:102606. doi: 10.1016/j.bbrep.2026.102606 (PMC13191627; doi:10.1016/j.bbrep.2026.102606)
Supplement: Multimedia component 1 [file mmc1.docx]

**Table S1** Details of Mouse qPCR primer sequences.

| Gene symbol |  | Primer sequence |
| --- | --- | --- |
| NQO1 | Forward primer | 5′- GAGAAGAGCCCTGATTGT-3′ |
|  | Reverse primer | 5′- AAAGGACCGTTGTCGTAC-3′ |
| NRF2 | Forward primer | 5′- AAAGCACAGCCAGCACATTC-3′ |
|  | Reverse primer | 5′-T GGGATTCACGCATAGGAGCA-3′ |
| HO-1 | Forward primer | 5′- CAGAACCCAGTCTATGCCCC-3′ |
|  | Reverse primer | 5′- GTGAGGCCCATACCAGAAGG-3′ |
| SOD2 | Forward primer | 5′- TGTGACTGCTGGAAAGGACG-3′ |
|  | Reverse primer | 5′- ATCCCAATCACTCCACAGGC-3′ |
| GAPDH | Forward primer | 5′- TGTTTCCTCGTCCCGTAG-3′ |
|  | Reverse primer | 5′- CAATCTCCACTTTGCCACT-3′ |

**Table S2** Immunohistochemistry (IHC) and Western blot (WB) antibodies

| Antibody | Catalog number and company | Dilution |
| --- | --- | --- |
| NRF2 | # ab76026 (Abcam) | IHC 1:100; WB 1:1000 |
| VEGFA | # ab46154(abcam) | IHC 1:100; WB 1:1000 |
| HO-1 | # 690002(zenbio) | IHC 1:100; WB 1:1000 |
| SOD2 | # R30160(zenbio) | IHC 1:100; WB 1:1000 |
| β-actin | #4970 (Cell Signaling Technology) | WB 1:1000 |
